# The Effect of Universal Influenza Immunization on Mortality and Health Care Use

**DOI:** 10.1371/journal.pmed.0050211

**Published:** 2008-10-28

**Authors:** Jeffrey C Kwong, Thérèse A Stukel, Jenny Lim, Allison J McGeer, Ross E. G Upshur, Helen Johansen, Christie Sambell, William W Thompson, Deva Thiruchelvam, Fawziah Marra, Lawrence W Svenson, Douglas G Manuel

**Affiliations:** 1 Institute for Clinical Evaluative Sciences, Toronto, Ontario, Canada; 2 Dalla Lana School of Public Health, University of Toronto, Toronto, Ontario, Canada; 3 Department of Family and Community Medicine, University of Toronto, Toronto, Ontario, Canada; 4 Department of Health Policy, Management and Evaluation, University of Toronto, Toronto, Ontario, Canada; 5 Department of Laboratory Medicine and Pathobiology, University of Toronto, Toronto, Ontario, Canada; 6 Mount Sinai Hospital, Department of Microbiology, Toronto, Ontario, Canada; 7 Sunnybrook Health Sciences Centre Primary Care Research Unit, Toronto, Ontario, Canada; 8 Health Information and Research Division, Statistics Canada, Ottawa, Ontario, Canada; 9 Health Statistics Division, Statistics Canada, Ottawa, Ontario, Canada; 10 Influenza Division**,** National Center for Immunization and Respiratory Diseases, Centers for Disease Control and Prevention, Atlanta, Georgia, United States of America; 11 British Columbia Centre for Disease Control, Vancouver, British Columbia, Canada; 12 Alberta Health and Wellness, Edmonton, Alberta, Canada; 13 Department of Public Health Sciences, University of Alberta, Edmonton, Alberta, Canada; 14 Department of Community Health Sciences, University of Calgary, Calgary, Alberta, Canada; The University of Hong Kong, Hong Kong

## Abstract

**Background:**

In 2000, Ontario, Canada, initiated a universal influenza immunization program (UIIP) to provide free influenza vaccines for the entire population aged 6 mo or older. Influenza immunization increased more rapidly in younger age groups in Ontario compared to other Canadian provinces, which all maintained targeted immunization programs. We evaluated the effect of Ontario's UIIP on influenza-associated mortality, hospitalizations, emergency department (ED) use, and visits to doctors' offices.

**Methods and Findings:**

Mortality and hospitalization data from 1997 to 2004 for all ten Canadian provinces were obtained from national datasets. Physician billing claims for visits to EDs and doctors' offices were obtained from provincial administrative datasets for four provinces with comprehensive data. Since outcomes coded as influenza are known to underestimate the true burden of influenza, we studied more broadly defined conditions. Hospitalizations, ED use, doctors' office visits for pneumonia and influenza, and all-cause mortality from 1997 to 2004 were modelled using Poisson regression, controlling for age, sex, province, influenza surveillance data, and temporal trends, and used to estimate the expected baseline outcome rates in the absence of influenza activity. The primary outcome was then defined as influenza-associated events, or the difference between the observed events and the expected baseline events. Changes in influenza-associated outcome rates before and after UIIP introduction in Ontario were compared to the corresponding changes in other provinces. After UIIP introduction, influenza-associated mortality decreased more in Ontario (relative rate [RR] = 0.26) than in other provinces (RR = 0.43) (ratio of RRs = 0.61, *p* = 0.002). Similar differences between Ontario and other provinces were observed for influenza-associated hospitalizations (RR = 0.25 versus 0.44, ratio of RRs = 0.58, *p* < 0.001), ED use (RR = 0.31 versus 0.69, ratio of RRs = 0.45, *p* < 0.001), and doctors' office visits (RR = 0.21 versus 0.52, ratio of RRs = 0.41, *p* < 0.001). Sensitivity analyses were carried out to assess consistency, specificity, and the presence of a dose-response relationship. Limitations of this study include the ecological study design, the nonspecific outcomes, difficulty in modeling baseline events, data quality and availability, and the inability to control for potentially important confounders.

**Conclusions:**

Compared to targeted programs in other provinces, introduction of universal vaccination in Ontario in 2000 was associated with relative reductions in influenza-associated mortality and health care use. The results of this large-scale natural experiment suggest that universal vaccination may be an effective public health measure for reducing the annual burden of influenza.

## Abstract 2

Comparing influenza-related mortality and health care use between Ontario and other Canadian provinces, Jeffrey Kwong and colleagues find evidence that Ontario's universal vaccination program has reduced the burden of influenza.

## Editors' Summary

**Background.:**

Seasonal outbreaks (epidemics) of influenza—a viral disease of the nose, throat, and airways—affect millions of people and kill about 500,000 individuals every year. These epidemics occur because of “antigenic drift”: small but frequent changes in the viral proteins to which the human immune system responds mean that an immune response produced one year by exposure to an influenza virus provides only partial protection against influenza the next year. Immunization can boost this natural immunity and reduce a person's chances of catching influenza. That is, an injection of killed influenza viruses can be used to prime the immune system so that it responds quickly and efficiently when exposed to live virus. However, because of antigenic drift, for influenza immunization to be effective, it has to be repeated annually with a vaccine that contains the major circulating strains of the influenza virus.

**Why Was This Study Done?:**

Public-health organizations recommend targeted vaccination programs, so that elderly people, infants, and chronically ill individuals—the people most likely to die from pneumonia and other complications of influenza—receive annual influenza vaccination. Some experts argue, however, that universal vaccination might provide populations with better protection from influenza, both directly by increasing the number of vaccinated people and indirectly through “herd immunity,” which occurs when a high proportion of the population is immune to an infectious disease, so that even unvaccinated people are unlikely to become infected (because infected people rarely come into contact with susceptible people). In this study, the researchers compare the effects of the world's first free universal influenza immunization program (UIIP), which started in 2000 in the Canadian province of Ontario, on influenza-associated deaths and health care use with the effects of targeted vaccine programs on the same outcomes elsewhere in Canada.

**What Did the Researchers Do and Find?:**

Using national records, the researchers collected data on influenza vaccination, on all deaths, and on hospitalizations for pneumonia and influenza in all Canadian provinces between 1997 and 2004. They also collected data on emergency department and doctors' office visits for pneumonia and influenza for Ontario, Quebec, Alberta, and Manitoba. They then used a mathematical model to estimate the baseline rates for these outcomes in the absence of influenza activity, and from these calculated weekly rates for deaths and health care use specifically resulting from influenza. In 1996–1997, 18% of the population was vaccinated against influenza in Ontario whereas in the other provinces combined the vaccination rate was 13%. On average, since 2000—the year in which UIIP was introduced in Ontario—vaccination rates have risen to 38% and 24% in Ontario and the other provinces, respectively. Since the introduction of UIIP, the researchers report, influenza-associated deaths have decreased by 74% in Ontario but by only 57% in the other provinces combined. Influenza-associated use of health care facilities has also decreased more in Ontario than in the other provinces over the same period.

**What Do These Findings Mean?:**

These findings are limited by some aspects of the study design. For example, they depend on the accuracy of the assumptions made when calculating events due specifically to influenza, and on the availability and accuracy of vaccination and clinical outcome data. In addition, it is possible that influenza-associated deaths and health care use may have decreased more in Ontario than in the other Canadian provinces because of some unrecognized health care changes specific to Ontario but unrelated to the introduction of universal influenza vaccination. Nevertheless, these findings indicate that, compared to the targeted vaccination programs in the other Canadian provinces, the Ontarian UIIP is associated with reductions in influenza-associated deaths and health care use, particularly in people younger than 65 years old. This effect is seen at a level of vaccination unlikely to produce herd immunity so might be more marked if the uptake of vaccination could be further increased. Thus, although it is possible that Canada is a special case, these findings suggest that universal influenza vaccination might be an effective way to reduce the global burden of influenza.

**Additional Information.:**

Please access these Web sites via the online version of this summary at http://dx.doi.org/10.1371/journal.pmed.0050211.

- Read the related *PLoSMedicine* Perspective by Cécile Viboud and Mark Miller
- A related *PLoSMedicine* Research Article by Carline van den Dool and colleagues is also available
- The Ontario Ministry of Health provides information on its universal influenza immunization program (in English and French)
- The World Health Organization provides information on influenza and on influenza vaccines (in several languages)
- The US Centers for Disease Control and Prevention provide information for patients and professionals on all aspects of influenza (in English and Spanish)
- MedlinePlus provides a list of links to other information about influenza (in English and Spanish)
- The UK National Health Service provides information about the science of immunization, including a simple explanatory animation of immunity

## Introduction

Annual epidemics of influenza continue to cause worldwide morbidity, mortality, and societal disruption. When there is good match to circulating strains, influenza vaccines are generally efficacious and cost-effective for most age groups [1–5]. However, some recent studies have questioned the validity of the nonexperimental evidence for influenza vaccine effectiveness in the elderly [6–9]. Efforts to reduce the impact of influenza have mainly involved targeted influenza immunization programs, which aim to vaccinate those at high risk of complications from influenza infection and their contacts [10,11].

In October 2000, the Canadian province of Ontario initiated the world's first large-scale universal influenza immunization program (UIIP) to provide free influenza vaccinations for the entire population ≥6 mo of age [12]. Supporters have argued that universal programs have several benefits: increased numbers of people directly protected from influenza by vaccination; possible indirect protection (i.e., protection extending to nonvaccinated individuals) arising from vaccinating greater proportions of the population; and having vaccine procurement and delivery systems in place for an influenza pandemic [13]. Critics have argued that uncertainties surrounding both the effectiveness and the cost-effectiveness of vaccinating healthy adults and children may not justify the costs of implementing such programs [14].

Ontario was the lone Canadian province to implement UIIP; other provinces maintained targeted programs. Canadian vaccination programs traditionally involve centralized vaccine procurement by provincial governments with publicly insured local delivery to high risk individuals and their close contacts/care providers. Vaccine is delivered in health care settings by nurses or physicians, or in community settings through public health departments. High risk individuals include seniors aged ≥65 y, individuals with chronic medical conditions, children aged 6–23 mo (since 2004), and pregnant women (since 2007) [11]. UIIP introduction in Ontario entailed expansion of prior vaccination activities, with local variations in delivery. Vaccine delivery settings include physician offices, hospitals, schools, workplaces, pharmacies, community centres, and shopping malls. The program also includes extensive media campaigns promoting the availability and benefits of free influenza vaccinations.

Our earlier work found that universal vaccination was associated with an overall incremental increase in vaccine uptake in the household population aged ≥12 y of 9 percentage points (24- versus 15-percentage-point increase in Ontario versus other provinces) between 1996–1997 and 2005 [15]. For those <65 y, greater increases over time were observed in Ontario compared to other provinces, whereas lesser increases were observed in Ontario compared to other provinces for those ≥85 y. All age groups in Ontario maintained higher uptake rates compared to corresponding age groups in other provinces throughout the study period.

In this study, we evaluated the effect of Ontario's UIIP on mortality, hospitalizations, emergency department (ED) use, and doctors' office visits compared to targeted programs in other Canadian provinces.

## Methods

### Study Population and Setting

This study included the population of the ten Canadian provinces from 1997 to 2004 who were eligible for universal, publicly insured health care services. Ethics approval was obtained from the Sunnybrook Health Sciences Centre Research Ethics Board, Toronto, Ontario.

### Study Design

We used a pre-/post-intervention study design with concurrent controls to assess the effect of Ontario's UIIP on influenza-associated mortality and health care use. Although ecological studies do not permit inference to individuals [16], this design was appropriate to evaluate the impact of a population-level vaccination program on population outcomes rather than the effect of vaccination on individual outcomes.

### Data Sources

Mortality data were obtained from Statistics Canada's Mortality Database, a national vital statistics dataset.

Hospitalization data were obtained from Statistics Canada's Hospital Morbidity Database, a national discharge abstract dataset.

Physician services data were obtained from the following provinces: Ontario, Quebec, Alberta, and Manitoba. These provinces account for approximately 76% of the Canadian population and were selected because of availability of comprehensive data on physician services.

Annual population estimates were obtained from Statistics Canada.

Vaccination rate data were obtained from the 1996–1997 cycle of the National Population Health Survey (NPHS) and the 2000–2001, 2003, and 2005 cycles of the Canadian Community Health Survey (CCHS). Conducted by Statistics Canada using telephone and in-person interviews, these surveys covered the household population aged ≥12 y but excluded members of the Canadian Forces, Indian reserves, and some remote areas, and those living in institutions. The surveys had response rates of between 79% and 85%; details have been described previously [15,17–21].

### Outcomes

Influenza outcomes are difficult to quantify because influenza infections are typically not confirmed. Since outcomes coded as influenza are known to underestimate the true burden of influenza, we studied more broadly defined conditions. These mortality and health care utilization outcomes vary according to a cyclical “baseline” function with winter peaks and summer troughs, but typically exhibit spikes during periods of influenza virus activity [22,23]. Our primary outcome was defined as influenza-associated outcomes, measured as the difference between observed events and expected baseline events during influenza seasons. This outcome reflects the excess number of events that occur because of influenza activity beyond what would be expected from background (baseline) rates. We chose this as our primary outcome because it is only these excess events that might be mitigated by a vaccination program.

For our primary mortality outcome, we included all deaths between 24 August 1997 and 14 August 2004 (primary study period comprising seven 52-wk periods).

We included all pneumonia and influenza (P&I) (ICD-9-CM 480–487, ICD-10-CA J10-J18) hospitalizations to acute care facilities during the study period, excluding admissions of nonresidents, transfers between institutions, and readmissions within 1 wk of discharge. Although discharge abstracts list up to 16 diagnoses, we used the first five codes to capture cases where influenza may have precipitated another condition requiring hospitalization (e.g., congestive heart failure) as well as nosocomial spread of influenza, while limiting the potential effects of differential coding practices between provinces and over time [24].

To assess ED use and visits to doctors' offices, we included all physician claims in ED and office settings during the study period for P&I. We included only one service claim per patient per physician per day, and where possible, we excluded claims that were associated with vaccination service codes (i.e., a visit to receive influenza vaccination).

### National Viral Surveillance Data

#### Respiratory virus detections.

A national network of hospital and provincial laboratories submit weekly reports of numbers of tests performed (using viral culture or direct antigen detection) and numbers of positive tests for influenza A, influenza B, and respiratory syncytial virus (RSV) to the Public Health Agency of Canada. The four Atlantic provinces were grouped owing to small numbers of specimens tested.

#### Predominance of influenza subtype A(H3N2).

A subset of laboratories report further case-specific data including subtype. Since A(H3N2)-predominant seasons have historically been more severe than A(H1N1)- and/or B-predominant seasons [23], these data were used to measure the proportion of the season's subtyped isolates that were influenza subtype A(H3N2) in each province and season (Table S1). This variable was necessary because weekly subtype-specific data were not available for the study period. Predominance of A(H3N2) was generally consistent across provinces.

#### Vaccine antigenic match.

A subset of viral isolates is sent to Canada's National Microbiology Laboratory for strain characterization. The sample was assumed to represent the distribution of strains for all influenza viruses circulating in the population. The percentage of circulating strains that did not match vaccine strains was calculated for each province and season (percent mismatch) (Table S1).

### Periods of Peak Influenza Activity

Periods of peak influenza activity were defined separately for each province and year, starting when the weekly percentage of tests positive for influenza was greater than 10% and ending when the percentage fell below that threshold for 2 consecutive wk (mean period duration = 11 wk) (Table S1).

### Statistical Analysis

We estimated influenza-associated events using a two-step procedure. We first ran multivariate regression models to estimate weekly events as a function of population and influenza season factors, and used this model to generate an expected baseline representing the pattern of events occurring in the absence of influenza. We then computed influenza-associated events as the difference between the number of observed events and expected baseline events during periods of influenza activity.

In the first step, we ran separate Poisson regression models for each outcome, according to province and age group (<5, 5–19, 20–49 [<50 for mortality], 50–64, 65–74, 75–84, 85+ y) [25]. We aggregated event counts by week and sex within province-age group combinations. The dependent variable was the weekly event count for males and females, and the offset parameter was the province-age group-sex population. Models controlled for sex; viral surveillance for influenza A, influenza B, and RSV; the seasonal percentage of A(H3N2) isolates; the percentage of mismatched strains; linear and quadratic terms to model annual trends; and sine and cosine terms with periods of 1 y to model seasonal fluctuations, as in previous studies [26,27]. To account for fluctuations in health service delivery during Christmas and post-Christmas holiday periods, categorical terms were included in the health care use models for these time periods. The model terms are outlined in Text S1. The expected baseline was generated by setting the influenza-related variables in the model (i.e., weekly percentage of tests positive for influenza A and B, seasonal percentage of A[H3N2] isolates) to zero as these were the terms that tracked influenza season peaks [26,27]. We incorporated variance overdispersion in the estimates of all standard errors (SE) to account for clustering of outcomes within weekly strata since outcomes to individuals within these strata may not be independent.

Weekly influenza-associated events were subsequently computed as the difference between the number of observed events and expected baseline events during periods of peak influenza activity, where expected counts were based on the adjusted Poisson models (Figure S1). These weekly estimates were aggregated to produce annual estimates. Overall and age-specific mean annual rates of influenza-associated outcomes were calculated for the periods before and after introduction of UIIP (pre-2000 versus post-2000), separately for Ontario and the other provinces combined.

Pre- and post-UIIP influenza-associated event rates were compared by dividing the adjusted postintervention rates by the preintervention rates to produce relative rates (RR) of UIIP effect separately for Ontario and the other provinces combined. The standard error of *ln*(RR) was computed assuming a Poisson distribution for the overall counts and incorporating the variance of the predicted baseline events [28]. The pre-/post-RRs for Ontario and other provinces combined were compared using the *z-*test and expressed as a ratio.

Model fit was evaluated by examining the standardized Pearson residuals for outlying points and secular trends. We also evaluated the presence of influential provinces by removing them individually from the model and reestimating the UIIP effect. Although the model did not optimally fit the extreme, short-lived spikes that occurred during the peaks of the influenza season, the fit during the remainder of the season was reasonable. We used the Durbin-Watson *d*-statistic to test for autocorrelation in the residuals, both including and excluding these influenza season spikes, averaging the correlations across province- and age group-specific models. Analyses were performed using SAS 9.1 (SAS Institute). All statistical tests were computed at the 5% level of significance and were two sided.

### Sensitivity Analyses

To confirm the robustness of the findings, several sensitivity analyses were conducted.

#### Tests of effect consistency.

To test the consistency of our findings, we used a 5% threshold for defining periods of peak influenza activity, leading to longer periods. We also lagged influenza and RSV activity forward or backward by 1 wk, in case of delays in viral testing or onset of serious illnesses (i.e., reports of positive tests and illness onset consistently occurring in different weeks).

To increase the number of influenza seasons in the study, we included data from the 1993–1994 to 1996–1997 influenza seasons, but this analysis excluded data from Quebec and the Atlantic provinces because prior to 1997–1998, the former did not report the number of tests performed, preventing calculation of weekly percentages of tests positive, and the latter performed too few tests (<25/wk during influenza season periods) to permit reliable calculation of percentages of tests positive.

As another test of consistency, we restricted the analysis to seasons in which A(H3N2) detections accounted for greater than 50% of the total isolates (A[H3N2]-predominant seasons).

For hospitalizations, we repeated the primary analysis using all 16 codes (any-listed) and using only the first code (primary-listed).

#### Tests of effect amplification.

For mortality, we examined deaths from respiratory and circulatory (R&C) conditions as a more specific outcome for influenza. Deaths from P&I were not assessed because of the small numbers of events at the province- and age-specific level, and because of a sharp decrease in P&I deaths coinciding with ICD-10 introduction in 2000 that is known to be a coding artifact [29].

The 2003–2004 influenza season was noteworthy for the emergence of the novel A/Fujian strain that accounted for 92% of the characterized isolates in Canada but was not included in that season's vaccine [30]. Since it was expected that vaccine efficacy would be reduced for that year, analyses were repeated excluding that season to assess the impact of universal vaccination for post-UIIP seasons with better vaccine match. We conducted another analysis that excluded all poor match seasons during the study period (i.e., the 1997–1998 A/Sydney season and the 2003–2004 A/Fujian season).

#### Tests of effect attenuation.

For the health care use outcomes, we examined the effect of UIIP on less specific outcomes. We examined hospitalizations for all respiratory conditions (ICD-9-CM 460–519, ICD-10-CA J00-J99), and for ED use and office visits, we examined selected respiratory conditions (composite of P&I, acute respiratory diseases [ARD] [ICD-9 460–466], otitis media [OM] [ICD-9 381–383] among those ≤50 y, and chronic obstructive pulmonary disease [COPD] [ICD-9 490–492, 496] among those aged ≥20 y) [31,32]. Higher-order sine and cosine terms were used in these models to achieve better fit to the observed seasonal variation in outcomes.

Data for the 2004–2005 season were available for hospitalizations, so a sensitivity analysis adding that year to the study period was performed. For that season, 35% of characterized isolates were A/California and 4% were B/Hong Kong strains, both of which were not included in that season's vaccine, although the A/Fujian component of the vaccine was believed to provide partial protection against A/California [33]. Therefore, as a moderately poor match season, it was expected to diminish the benefits of the UIIP.

#### Tests of effect specificity.

To assess the specificity of our findings, we examined rates of all observed events, as opposed to influenza-associated events, during the month of July, since influenza viruses are not circulating in the summer and we would not expect any influenza-associated events nor any benefit from influenza vaccination.

As another test of specificity, we examined the effect of the UIIP on selected control conditions, by computing RRs for all events during the month of February, when influenza viruses are almost always circulating. For mortality, we used all non-R&C conditions, for hospitalizations, we examined hernia, and for ED use and office visits, we considered lacerations.

#### Dose-response relationship.

Regions with greater increases in vaccination rates are expected to have greater decreases in influenza-associated outcomes. To assess the presence of such a relationship, we plotted age group-, province-, and outcome-specific RRs for mean influenza-associated events against absolute changes in vaccination rates. We used the mean of the estimates from the three cycles of the Canadian Community Health Survey as the measure of post-UIIP vaccine uptake and the 1996–1997 National Population Health Survey estimate as the measure of pre-UIIP vaccine uptake. To match with the age groupings of the outcomes data, we assumed that the vaccination rates of those aged 12 to 19 y applied to children as young as 5 y. We fitted weighted linear models separately by outcome and age group (<65 y and ≥65 y), weighting by the inverse of the variance of the RRs.

## Results

### Descriptive Results

**Figure 1:**
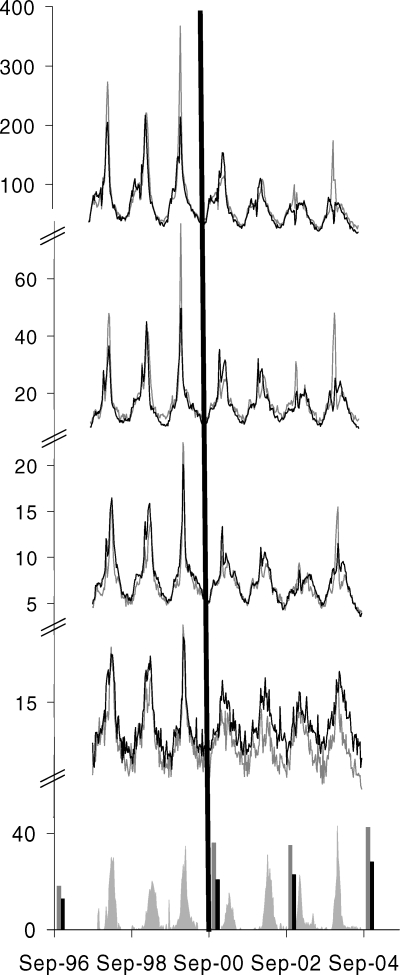
Study Outcome Rates over the Study Period Demonstrating Year-to-Year Variability in Mortality and Health Care Use, Temporal Correlation with Ontario Influenza Viral Surveillance Data, and Increasing Influenza Vaccination Rates Demonstrating Greater Increases in Ontario Compared to Other Provinces Combined Event rates (from top to bottom) for doctors' office visits, ED use, hospitalizations, and mortality (grey lines are for Ontario and black lines are for other provinces combined) are expressed as rates per 100,000 on the upper sections of the vertical axis. Viral surveillance data (grey shaded areas) are expressed as the weekly percentage of tests positive on the lower section of the vertical axis. Vaccination rates for the household population aged ≥12 y (grey vertical bars are for Ontario and black vertical bars are for other provinces combined) are expressed as the percentage of the population vaccinated on the lower section of the vertical axis. The horizontal axis represents time. The black vertical line represents UIIP introduction.

Over the study period, mean annual outcome rates were highest in older people and young children (Table 1). Plots of weekly outcome rates over the study period for Ontario and other provinces demonstrated seasonal trends with spikes during periods of influenza activity (Figure 1).

**Table 1:**
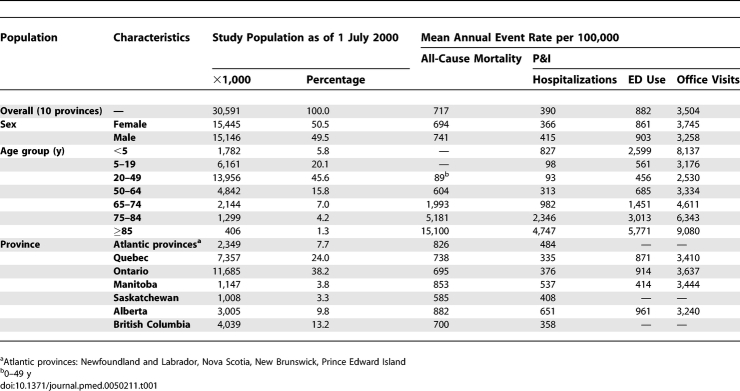
Study Population Demographics and Mean Annual Event Rates over Study Period

### Vaccination Rates

Between the pre-UIIP 1996–1997 estimate to the mean post-UIIP vaccination rate, influenza vaccination rates for the household population aged ≥12 y increased 20 percentage points (18%–38%) for Ontario, compared to 11 percentage points (13%–24%) for other provinces (*p* < 0.001) (Table 2). For those <65 y, the vaccination rate increases were greater in Ontario than in other provinces, while for those ≥75 y, the increase was smaller in Ontario. For all age groups, Ontario always achieved higher vaccination rates than other provinces.

**Table 2:**
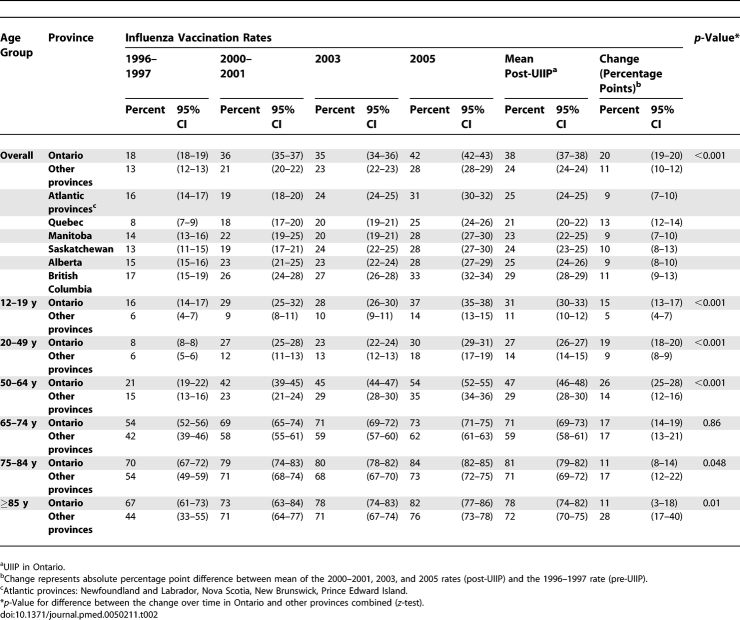
Influenza Vaccination Rates over Time for Ontario and Other Provinces

### Influenza-Associated Mortality and Health Care Use

After UIIP introduction, influenza-associated mortality for the overall population decreased 74% in Ontario (RR = 0.26, 95% confidence interval [CI], 0.20–0.34) compared to 57% in other provinces (RR = 0.43, 95% CI, 0.37–0.50) (ratio of RRs = 0.61, *p* = 0.002) (Table 3). In age-specific analyses, larger mortality decreases in Ontario were found to be statistically significant only in those ≥85 y.

**Table 3:**
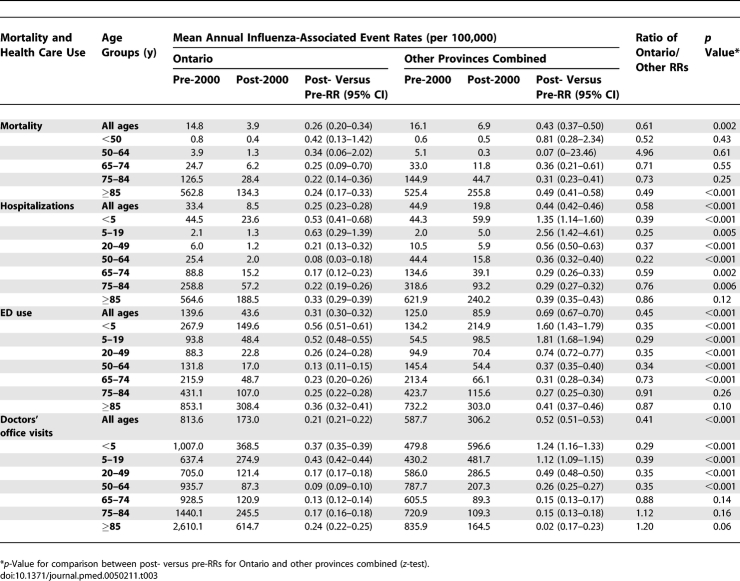
Effect of UIIP on Influenza-Associated Mortality and Health Care Use Rates

Overall, influenza-associated health care use decreased more in Ontario than other provinces for hospitalizations (RR = 0.25 versus 0.44, ratio of RRs = 0.58, *p* < 0.001), ED use (RR = 0.31 versus 0.70, ratio of RRs = 0.45, *p* < 0.001), and doctors' office visits (RR = 0.21 versus 0.53, ratio of RRs = 0.41, *p* < 0.001). In age-specific analyses, greater decreases were consistently observed in Ontario than other provinces for age groups <65 y. For seniors, greater decreases were observed in Ontario than other provinces for hospitalizations among those aged 65–84 y and for ED use among those 65–74 y.

There were no patterns detected in the standardized residual plots against time, and no evidence of influential provinces (Table S2). A greater than expected number of outliers occurred during the 5 wk each year that influenza activity was most intense, however, since the main purpose of the model was to estimate the baseline rate in the absence of influenza activity, these were not of major concern. The mean first order autocorrelation coefficients (*r*) including (excluding) influenza spikes were 0.051 (0.039) for mortality, 0.255 (0.209) for hospitalizations, 0.398 (0.312) for ED use, and 0.548 (0.492) for doctors' office visits.

### Sensitivity Analyses

The sensitivity analyses generally produced similar RR ratios as the primary analysis for tests of consistency, smaller RR ratios for tests of amplification, larger RR ratios for tests of attenuation, and RR ratios approximately equal to 1 for tests of specificity, as expected (Table 4). Some exceptions were noted, in particular mortality for R&C conditions, which was not increased as anticipated; the secondary outcomes for ED use and office visits (selected respiratory conditions), which were not attenuated as anticipated; and the analysis for health care use including only A(H3N2) seasons resulted in attenuation rather than consistency compared to the primary analysis.

**Table 4:**
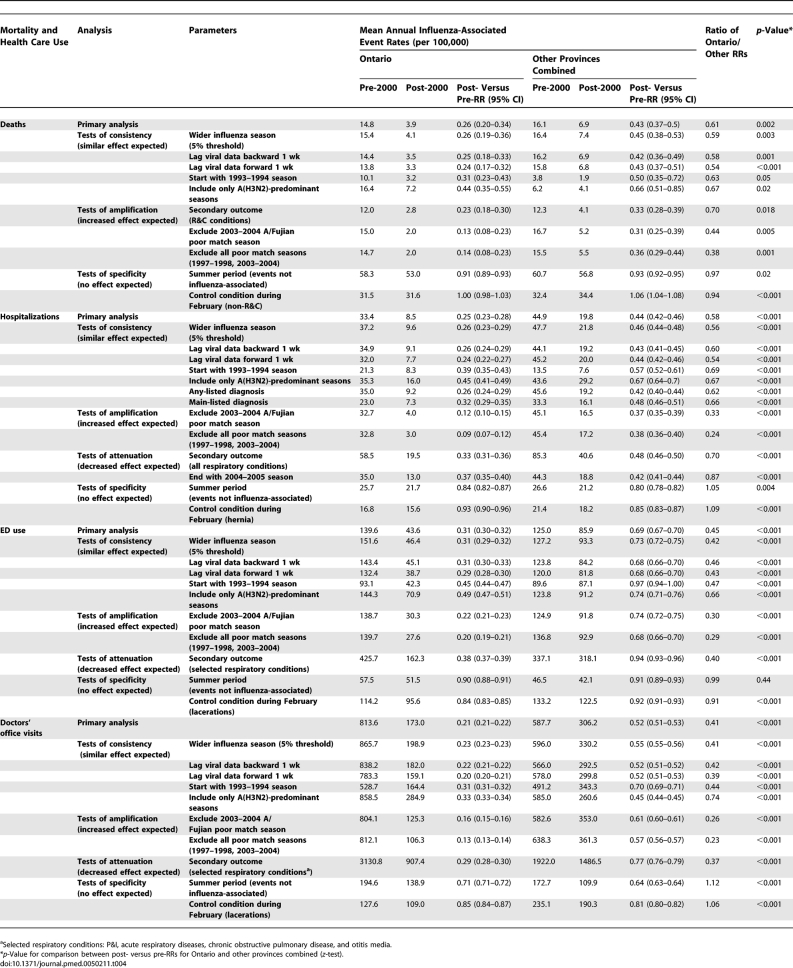
Sensitivity Analyses

**Figure 2:**
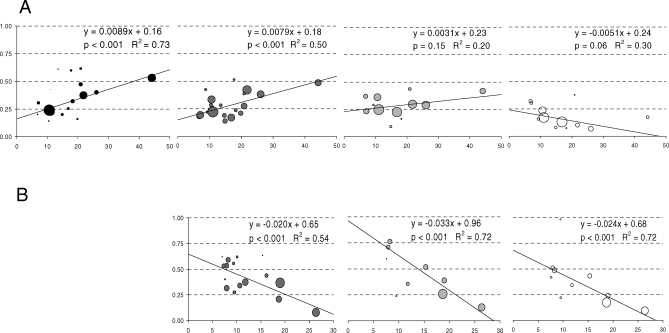
Sensitivity Analysis to Assess the Presence of a Dose-Response Relationship in Age Groups Older (A) and Younger (B) than 65 y for (from Left to Right) Mortality [(A) only], Hospitalizations, ED Use, and Doctors' Office Visits The vertical axis represents the age group-, province-, and outcome-specific post- versus pre-2000 RRs. The horizontal axis represents the absolute post- versus pre-2000 change in influenza vaccination rates (%). The bubble sizes represent the inverse of the variances of the post- versus pre-2000 RRs. The *p-*values correspond to the significance of the estimates for the slope of the lines from weighted linear regression models (*t-*tests).

We found a dose-response relationship where greater increases in vaccine uptake were associated with greater decreases in influenza-associated outcomes for all health care use outcomes for age groups <65 y (all slopes negative, *p* < 0.001) (Figure 2). For the elderly, the opposite relationship was observed for mortality and hospitalizations, and no relationship was noted for ED use and office visits.

## Discussion

After introduction of the world's first large-scale UIIP, we found that influenza-associated mortality, hospitalizations, ED use, and office visits decreased more overall in Ontario compared with other provinces. Age-specific analyses revealed greater temporal drops in health care use in Ontario compared to other provinces for younger age groups (i.e., ratios of pre-/post-RRs for Ontario versus other provinces being closer to zero), with the gap between the RRs narrowing with increasing age (i.e., ratios of RRs being closer to one). This is consistent with the age-specific pattern for temporal changes in vaccine uptake, with greater increases over time in Ontario compared to other provinces for younger age groups leading to greater expectations of benefits from the UIIP. However, for older age groups, in particular those ≥75 y, greater incremental increases in vaccine uptake over time were observed in other provinces compared to Ontario, yet among all the outcomes, none of the RR ratios were greater than one. This result is contrary to what one would expect based on the assumption that greater increases in vaccine uptake in those populations should have been associated with greater decreases in outcome rates, and therefore suggests that influenza vaccines may not be as effective in reducing mortality and health care use in older people compared to younger age groups.

Sensitivity analyses demonstrated consistency, specificity, and the presence of a dose-response relationship between temporal changes in vaccination rates and outcomes among those <65 y. A limited number of inconsistent results warrant discussion. While the analysis restricted to A(H3N2)-predominant seasons was expected to be a test of consistency, the results for the three health care use outcomes suggested effect attenuation. This was likely because that analysis over-weighted the influence of the 2003–2004 poor match season by excluding the 2000–2001 and 2002–2003 non-A(H3N2)-predominant seasons. Other unexpected results were the lack of attenuation when examining the less specific secondary outcomes for ED use and office visits. This may have been because the selected conditions are nearly as specific to influenza as the primary outcome. Another inconsistency was the lack of amplification for the supposedly more specific secondary outcome for mortality, respiratory, and circulatory conditions. This lack of amplification may be because R&C mortality accounts for the bulk of the mortality from all causes [26,34]. Regardless, the totality of these results suggest that introduction of universal influenza vaccination in Ontario has been associated with incremental benefits in terms of mortality and health care use compared to targeted programs.

A previous study reported that UIIP introduction did not lead to reduced influenza incidence [35], but used numbers of laboratory-confirmed cases as the outcome. Their negative finding may have been a result of ascertainment bias arising from a 10-fold increase in testing for influenza over the study period [36].

Although many studies have found that for years with good vaccine match, influenza vaccines are effective in preventing hospitalizations and mortality in the elderly [2,3], the true extent of vaccine effectiveness versus the effects of healthy user biases has been the subject of much recent debate [6–9]. This study does not resolve that debate, however, the findings suggest that in elderly populations, increasing vaccination levels were not associated with decreases in influenza-associated outcomes, and may have even been associated with increased risk of mortality and hospitalizations. Potential explanations include immune senescence, or a decline in immune responsiveness with advancing age [37–40], and uncontrolled confounding. In working age adults, the effectiveness of influenza vaccines for preventing influenza infection and health care use is less controversial, and the results from this study are consistent with this literature [4,5]. While previous studies have failed to demonstrate reductions in hospitalizations among working age adults [41,42] and in health care use among children [5], the findings from this study suggest that increasing population-level vaccine uptake in these age groups may be associated with substantial reductions in these outcomes.

Despite universal availability of publicly insured health care services, enhanced access to free influenza vaccines in Ontario since 2000, and extensive media communications, only an estimated average of 38% of the overall household population reported receiving them during the post-UIIP introduction period. Although vaccine uptake was higher in older age groups (e.g., approximately 80% among those ≥75 y), it is uncertain that levels required for appreciable herd immunity effects were obtained in the overall population, particularly in younger age groups. Several studies have claimed the existence of indirect benefits arising from vaccinating children, including a report on the impact of vaccinating a community's children on illness rates in the adult population during the 1968 pandemic [43], a retrospective ecological analysis of a mass vaccination program for schoolchildren and trends in seniors' mortality in Japan [44], and a clinical trial that reported decreased rates of visits for acute respiratory infections among adults after vaccinating children with live-attenuated influenza vaccines [45]. Furthermore, a modeling study of the potential effects of vaccinating children on outcomes in the population suggested that increasing vaccination rates of children from 20% to 40% would lead to approximately 50% reductions in cases of influenza, hospitalizations, and deaths [46]. In Ontario, vaccination rates among those aged 12 to 19 y increased from 16% to 31% over the study period, while in other provinces, rates increased from 6% to 11%. We observed more than 50% reductions in both influenza-associated mortality and health care use over the study period among seniors in both Ontario and other provinces. The bulk of these decreases are likely attributable to less severe influenza seasons across Canada in the postintervention period, but the larger observed decreases in mortality for seniors aged ≥85 y, hospitalizations for seniors aged 65–84 y, and ED use for those aged 65–74 y in Ontario compared to other provinces may be compatible with some indirect benefits as previously posited [46]. Unfortunately, this study does not permit determination of the direct versus indirect benefits of universal vaccination.

Strengths of the study include the unique opportunity for a “natural experiment” and the fact that size of the populations of Ontario and the other provinces combined are comparable. We addressed most of the criteria outlined recently in a framework for addressing residual bias by Simonsen et al., including: the selection of more specific outcomes for the primary study outcomes (with the exception of all-cause mortality) and the use of consistent modeling techniques and laboratory surveillance data to estimate influenza-associated events to reduce the potential dilution of benefits from universal vaccination that might arise from employing all influenza season-associated events as the outcome measure (end-point specificity); the sensitivity analyses with poor match seasons excluded (vaccine match); and the inclusion of events only during periods of peak influenza activity and the sensitivity analysis using a summer period (seasonality) [8]. Lastly, it is notable that the results are consistent across three distinct outcomes (hospitalizations, ED use, and office visits), reducing the likelihood of spurious findings attributable to data quality or other biases.

This study has a number of limitations. Individual-level vaccination and outcome data were not available, necessitating an ecological study. However, use of this design is appropriate for assessing the public health impact of a population-wide intervention. As with other influenza studies using health databases, the selected outcomes are nonspecific and may be due to causes other than influenza, but the strategies described above partly address this issue. The quality and reliability of the outcome data over time, for multiple jurisdictions, and across different classification systems (i.e., ICD-9 versus ICD-10) remain uncertain. The validity of statistical models to estimate influenza-associated events is limited by uncertainty of their accuracy, in spite of our best efforts to achieve optimal model fit. Laboratory viral surveillance data are potentially susceptible to ascertainment and reporting biases; the weekly proportion of tests positive is felt to be the most robust measure of viral activity. Another drawback of the study is that no vaccination rate data are available for those <12 y of age, an age group that experiences particularly high rates of less severe influenza-associated outcomes, or for institutionalized seniors, a group that experiences higher rates of more severe outcomes. Relatively high residual autocorrelation was likely due to the difficulty in modeling a baseline influenza rate that did not display purely cyclical behaviour despite inclusion of higher order Fourier series terms; however, it suggests the possibility of residual confounding due to season-specific factors such as temperature or relative humidity. We were also not able to include other potential confounders such as strain-specific influenza surveillance data, prevalence of individual comorbidities, socioeconomic status, smoking rates, polysaccharide or conjugated pneumococcal vaccination, antiviral medication use, and provincial health care system capacity, but we have no reason to believe that these factors changed more over time in Ontario compared to other provinces. For example, pneumococcal vaccination of children has been shown to reduce rates of pneumonia-related admissions in older age groups [47,48] and might have contributed to temporal changes in health care use for pneumonia, but Ontario's infant pneumococcal vaccination program was introduced in January 2005, subsequent to the introduction of pneumococcal vaccination programs in four other provinces (September 2002 in Alberta, September 2003 in British Columbia, October 2004 in Manitoba, and December 2004 in Quebec) (personal correspondence, A.-M. Frescura, Public Health Agency of Canada). Therefore the greater drops in influenza-associated outcomes observed in Ontario after 2000 are unlikely to be attributable to pneumococcal vaccination. Because of the observational nature of our study, we cannot rule out the possibility of “healthy population” bias (i.e., healthier populations are more likely to get vaccinated and have better outcomes), but our study design and various tests of specificity support the absence of such a bias. Most of the limitations arise from the necessity of using existing health data. As we are not aware of other data or approaches that can resolve these limitations, we believe this study represents the best possible evaluation of a real-world natural experiment that Ontario's unique strategy to offer influenza vaccination to the entire population represents.

Despite these limitations, this study provides suggestive evidence of the population-based effectiveness of universal vaccination programs using inactivated influenza vaccines. It is not possible to definitively declare superiority of universal programs over targeted programs, as the findings from this study may not generalize to other settings. But by reducing financial barriers and increasing awareness and accessibility, universal vaccination may be an effective strategy for increasing a population's protection against influenza. Future studies to develop more immunogenic influenza vaccines, to test novel strategies for further increasing vaccine uptake, and to examine the cost-effectiveness of universal influenza vaccination may be valuable.

## Supporting Information

Figure S1 Estimating Influenza-Associated Events, Using P&I Hospitalizations for Ontario Females ≥85 y as an Example The left vertical axis represents hospitalization rate per 100,000. The horizontal axis represents time. The solid black line represents observed hospitalizations, the dashed black line represents baseline hospitalizations in the hypothetical absence of influenza, and the black vertical bars represent influenza-associated hospitalizations (observed hospitalizations minus baseline hospitalizations). Viral surveillance data (grey shaded areas) are expressed as the weekly percentage of tests positive on the right vertical axis. The grey dashed vertical lines denote periods of peak influenza activity.
(539 KB PPT)

Table S1 Duration of Periods of Peak Influenza Activity, Influenza A(H3N2) Predominance, and Vaccine Antigenic Mismatch between Circulating and Vaccine Strains, by Influenza Season and Province (97 KB DOC)

Table S2 Analysis to Evaluate the Presence of Influential Provinces by Removing Them Individually from the Model (74 KB DOC)

Text S1 Description of the Multivariate Poisson Regression Model (43 KB DOC)

## Abbreviations

CI: confidence interval
ED: emergency department
P&I: pneumonia and influenza
R&C: respiratory and circulatory
RR: relative rate
UIIP: universal influenza immunization program
