# Supplementary figures and images for: The Effect of Universal Influenza Immunization on Mortality and Health Care Use
Source: PLoS Med. 2008 Oct 28;5(10):e211. doi: 10.1371/journal.pmed.0050211 (PMC2573914; doi:10.1371/journal.pmed.0050211)

## Slide 1
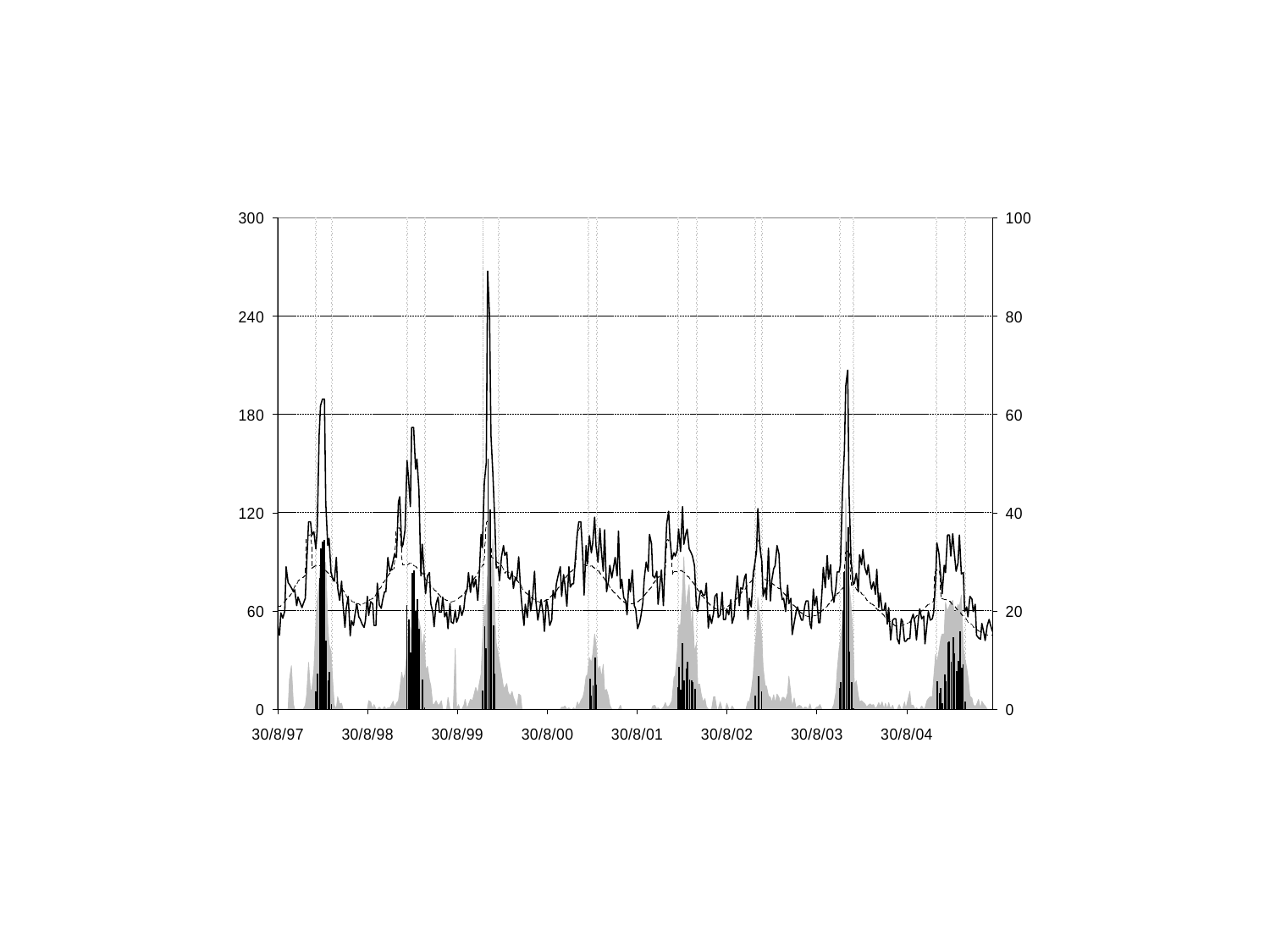

Supplement: Figure S1 — The left vertical axis represents hospitalization rate per 100,000. The horizontal axis represents time. The solid black line represents observed hospitalizations, the dashed black line represents baseline hospitalizations in the hypothetical absence of influenza, and the black vertical bars represent influenza-associated hospitalizations (observed hospitalizations minus baseline hospitalizations). Viral surveillance data (grey shaded areas) are expressed as the weekly percentage of tests positive on the right vertical axis. The grey dashed vertical lines denote periods of peak influenza activity. (539 KB PPT) [file pmed.0050211.sg001.ppt]
