# Supplementary material for: The Effect of Universal Influenza Immunization on Mortality and Health Care Use
Source: PLoS Med. 2008 Oct 28;5(10):e211. doi: 10.1371/journal.pmed.0050211 (PMC2573914; doi:10.1371/journal.pmed.0050211)
Supplement: Table S1 — (97 KB DOC) [file pmed.0050211.st001.doc]

**Table S1. Duration of periods of peak influenza activity, influenza A(H3N2) predominance, and vaccine antigenic mismatch between circulating and vaccine strains, by influenza season and province**

|  |  |  |  |  | **Primary study period** | | | | | | |  |
| --- | --- | --- | --- | --- | --- | --- | --- | --- | --- | --- | --- | --- |
|  |  |  |  |  |  |  |  | **Universal vaccination in Ontario** | | | |  |
|  | **1993-1994** | **1994-1995** | **1995-1996** | **1996-1997** | **1997-1998** | **1998-1999** | **1999-2000** | **2000-2001** | **2001-2002** | **2002-2003** | **2003-2004** | **2004-2005** |
| **Duration of periods of peak influenza activity (weeks)** | | | | | | | | | | | | |
| Atlantic provinces† | 7 | 5 | 10 | 9 | 8 | 11 | 16 | 9 | 17 | 10 | 13 | 13 |
| Quebec | - | - | - | - | 15 | 9 | 15 | 10 | 11 | 11 | 11 | 11 |
| Ontario | 4 | 7 | 8 | 8 | 10 | 11 | 10 | 5 | 12 | 5 | 9 | 18 |
| Manitoba | 6 | 4 | 6 | 7 | 7 | 6 | 9 | 7 | 9 | 11 | 5 | 9 |
| Saskatchewan | 3 | 5 | 6 | 10 | 6 | 15 | 10 | 14 | 10 | 9 | 14 | 10 |
| Alberta | 7 | 4 | 8 | 4 | 9 | 10 | 7 | 11 | 11 | 6 | 9 | 9 |
| British Columbia | 6 | 10 | 7 | 16 | 14 | 10 | 14 | 16 | 18 | 16 | 11 | 21 |
| **Percent A(H3N2) isolates (%)** | | | | | | | | | | | | |
| Atlantic provinces† | 73 | 75 | 0 | 69 | 100 | 95 | 100 | 0 | 98 | 0 | 98 | 96 |
| Quebec | 100 | 60 | 0 | 55 | 100 | 95 | 67 | 0 | 97 | 8 | 99 | 90 |
| Ontario | 99 | 80 | 8 | 78 | 73 | 72 | 93 | 1 | 64 | 2 | 99 | 75 |
| Manitoba | 92 | 74 | 10 | 55 | 100 | 88 | 99 | 0 | 59 | 0 | 99 | 68 |
| Saskatchewan | 99 | 64 | 1 | 62 | 100 | 82 | 92 | 0 | 65 | 3 | 100 | 93 |
| Alberta | 100 | 71 | 9 | 54 | 100 | 93 | 95 | 0 | 91 | 16 | 100 | 86 |
| British Columbia | 100 | 70 | 32 | 49 | 98 | 92 | 98 | 0 | 90 | 3 | 100 | 89 |
| **Percent mismatch between circulating and vaccine strains (%)** | | | | | | | | | | | | |
| Atlantic provinces† | 0 | 55 | 0 | 0 | 75 | 0 | 0 | 0 | 2 | 0 | 96 | 44 |
| Quebec | 4 | 65 | 0 | 0 | 56 | 0 | 32 | 1 | 2 | 0 | 100 | 30 |
| Ontario | 2 | 26 | 1 | 0 | 65 | 0 | 7 | 0 | 35 | 0 | 100 | 42 |
| Manitoba | 0 | 63 | 5 | 0 | 100 | 0 | 0 | 0 | 0 | 0 | 99 | 23 |
| Saskatchewan | 0 | 28 | 0 | 0 | 90 | 0 | 2 | 0 | 2 | 0 | 82 | 47 |
| Alberta | 8 | 29 | 0 | 0 | 84 | 0 | 2 | 0 | 9 | 0 | 92 | 33 |
| British Columbia | 0 | 46 | 0 | 0 | 88 | 0 | 0 | 0 | 9 | 0 | 96 | 29 |

†Atlantic provinces = Newfoundland and Labrador, Nova Scotia, New Brunswick, Prince Edward Island
