# Supplementary material for: The Effect of Universal Influenza Immunization on Mortality and Health Care Use
Source: PLoS Med. 2008 Oct 28;5(10):e211. doi: 10.1371/journal.pmed.0050211 (PMC2573914; doi:10.1371/journal.pmed.0050211)
Supplement: Table S2 — (74 KB DOC) [file pmed.0050211.st002.doc]

**Table S2. Sensitivity analysis to assess the presence of influential provinces by removing one province at a time**

|  | Mean annual influenza-associated event rates (per 100,000) | | | | | | Ratio of |  |
| --- | --- | --- | --- | --- | --- | --- | --- | --- |
|  | Ontario | | | Other provinces combined | | | Ontario/ |  |
|  | Pre-2000 | Post-2000 | Post vs. pre  RR† (95% CI) | Pre-2000 | Post-2000 | Post vs. pre  RR† (95% CI) | Other  RRs | p value* |
| **Deaths** |  |  |  |  |  |  |  |  |
| *Primary analysis (no provinces excluded)* | 14.8 | 3.9 | 0.26 (0.20-0.34) | 16.1 | 6.9 | 0.43 (0.37-0.50) | 0.61 | 0.002 |
| Atlantic provinces excluded |  |  |  | 16.9 | 7.7 | 0.45 (0.39-0.53) | 0.57 | <0.001 |
| Quebec excluded |  |  |  | 12.0 | 4.3 | 0.36 (0.26-0.48) | 0.73 | 0.13 |
| Manitoba excluded |  |  |  | 15.7 | 6.9 | 0.44 (0.38-0.52) | 0.59 | <0.001 |
| Saskatchewan excluded |  |  |  | 16.1 | 6.7 | 0.42 (0.36-0.49) | 0.62 | 0.003 |
| Alberta excluded |  |  |  | 16.8 | 7.3 | 0.44 (0.37-0.51) | 0.60 | 0.001 |
| British Columbia excluded |  |  |  | 18.0 | 7.6 | 0.42 (0.36-0.49) | 0.62 | 0.002 |
|  |  |  |  |  |  |  |  |  |
| **Hospitalizations** |  |  |  |  |  |  |  |  |
| *Primary analysis (no provinces excluded)* | 33.4 | 8.5 | 0.25 (0.23-0.28) | 44.9 | 19.8 | 0.44 (0.42-0.46) | 0.58 | <0.001 |
| Atlantic provinces excluded |  |  |  | 43.9 | 20.0 | 0.46 (0.43-0.48) | 0.56 | <0.001 |
| Quebec excluded |  |  |  | 47.6 | 18.0 | 0.38 (0.36-0.40) | 0.67 | <0.001 |
| Manitoba excluded |  |  |  | 44.6 | 20.4 | 0.46 (0.44-0.48) | 0.56 | <0.001 |
| Saskatchewan excluded |  |  |  | 43.5 | 18.7 | 0.43 (0.41-0.45) | 0.59 | <0.001 |
| Alberta excluded |  |  |  | 43.3 | 19.9 | 0.46 (0.44-0.48) | 0.55 | <0.001 |
| British Columbia excluded |  |  |  | 48.0 | 21.8 | 0.45 (0.43-0.48) | 0.56 | <0.001 |
|  |  |  |  |  |  |  |  |  |
| **Emergency department use** |  |  |  |  |  |  |  |  |
| *Primary analysis (no provinces excluded)* | 139.6 | 43.6 | 0.31 (0.3-0.32) | 125.0 | 85.9 | 0.69 (0.67-0.70) | 0.45 | <0.001 |
| Quebec excluded |  |  |  | 125.9 | 89.2 | 0.71 (0.68-0.73) | 0.44 | <0.001 |
| Manitoba excluded |  |  |  | 134.0 | 92.3 | 0.69 (0.67-0.71) | 0.45 | <0.001 |
| Alberta excluded |  |  |  | 113.7 | 76.4 | 0.67 (0.65-0.69) | 0.46 | <0.001 |
|  |  |  |  |  |  |  |  |  |
| **Doctors’ office visits** |  |  |  |  |  |  |  |  |
| *Primary analysis (no provinces excluded)* | 813.6 | 173.0 | 0.21 (0.21-0.22) | 587.7 | 306.2 | 0.52 (0.51-0.53) | 0.41 | <0.001 |
| Quebec excluded |  |  |  | 649.9 | 353.1 | 0.54 (0.53-0.55) | 0.39 | <0.001 |
| Manitoba excluded |  |  |  | 595.0 | 314.3 | 0.53 (0.52-0.54) | 0.40 | <0.001 |
| Alberta excluded |  |  |  | 549.1 | 273.2 | 0.50 (0.49-0.51) | 0.43 | <0.001 |

† Relative rate. * p value for comparison between post vs. pre relative rates for Ontario and other provinces combined (z-test).
