# Supplementary material for: The Effect of Universal Influenza Immunization on Mortality and Health Care Use
Source: PLoS Med. 2008 Oct 28;5(10):e211. doi: 10.1371/journal.pmed.0050211 (PMC2573914; doi:10.1371/journal.pmed.0050211)
Supplement: Text S1 — (43 KB DOC) [file pmed.0050211.sd001.doc]

**Text S1. Description of the multivariate Poisson regression model.**

The multivariate Poisson regression model was expressed as follows:

*ln*(Y) = *ln*(population) + β_0_ + β_1_[sex] + β_2_[%FluA] + β_3_[%FluB] + β_4_[%RSV] + β_5_[%A(H3N2)] + β_6_[%mismatch] + β_7_[t] + β_8_[t^2^] + β_9_[sin(2tπ/52)] + β_10_[cos(2tπ/52)] + ε

Y represents the weekly number of events for a particular outcome (e.g., all-cause mortality) in a province for a specific age group and sex stratum. The offset term is the log of the annual province-, age- and sex-specific population size. β_0_ is the intercept and β_1_ estimates the effects of sex. β_2_ through β_4_ account for the weekly percentage of provincial specimens testing positive for influenza A, influenza B, and RSV, respectively. β_5_ accounts for the percentage of A(H3N2) isolates and β_6_ accounts for the percentage of circulating strains mismatched to vaccine strains in a season. β_7_ and β_8_ are the coefficients for the linear and quadratic time trend terms, with t expressed as the week since August 24, 1997 (1 to 416) divided by 52. β_9_ and β_10_ account for the seasonal cyclical pattern. We used a period of 1 year, as in previous studies.[24,25] The error term ε represents random error in the model. Additional terms for Christmas holiday weeks and the post-Christmas holiday week were included in the health care use models to account for fluctuations in health care service delivery during holiday and post-holiday periods.
